# Physicochemical, Thermal, and Morphological Properties of Chitosan Nanoparticles Produced by Ionic Gelation

**DOI:** 10.3390/foods11233841

**Published:** 2022-11-28

**Authors:** Elham Alehosseini, Hoda Shahiri Tabarestani, Mohammad Saeed Kharazmi, Seid Mahdi Jafari

**Affiliations:** 1 Faculty of Food Science and Technology, Gorgan University of Agricultural Sciences and Natural Resources, Gorgan 4918943464, Iran; 2 Faculty of Medicine, University of California, Riverside, CA 92679, USA

**Keywords:** chitosan nanoparticles, ionic gelation, thermal analysis, morphology

## Abstract

Chitosan nanoparticles (CSNPs) can be widely used in the food, pharmaceutical, and cosmetic sectors due to their high performance, unique properties, and high surface area. In this research, CSNPs were produced by the ionic gelation method and using sodium tripolyphosphate (STPP) as an appropriate technique compared to the conventional methods. To evaluate the effects of various factors on the size, zeta potential (ZP), and optimal synthesis conditions, different concentrations of CS (1, 3, and 5 mg/mL), STPP (0.5, 0.75, and 1 mg/mL), and CS to STPP ratio (1:1, 3:1, and 5:1) were applied and optimized using the response surface methodology. The size of CSNPs was increased by using higher concentrations of CS, STPP, and CS/STPP ratios. The value of ZP was determined positive and it increased with increasing CS concentrations and CS/STPP ratios. ATR-FTIR spectra revealed interactions between CS and STPP. The DSC thermogram of CSNPs showed a double sharp endothermic peak at about 74.5 °C (ΔH = 122.00 J/g); further, the TGA thermograms indicated the total weight loss of STPP, CS, and CSNPs as nearly 3.30%, 63.60%, and 52.00%, respectively. The XRD data also revealed a greater chain alignment in the CSNPs. Optimized, the CSNPs can be used as promising carriers for bioactive compounds where they also act as efficient stabilizers in Pickering emulsions.

## 1. Introduction

Chitosan (CS) is known as the second most plentiful natural biopolymer (β-(1→4)-linked N-acetyl-D-glucosamine and D-glucosamine), obtained by the alkaline deacetylation of chitin, which has biodegradable, biocompatible, bioadhesive, emulsifying, prebiotic, intrinsic antimicrobial, antioxidant, and nontoxic properties [1,2,3,4,5]. Since CS has been introduced as a safe substance by the Food and Drug Administration (FDA), with good solubility in an aqueous acidic solution, it can be considered a potential candidate for both food and biomedical applications; hence, CS is used in various forms such as nano/microparticles, films, gels, beads, fibers, etc. [6]. Moreover, due to the physicochemical, biological, and antimicrobial properties of CS nanoparticles (CSNPs), they have a wide range of applications such as in tissue engineering, cancer diagnosis, drug delivery, enzyme immobilization, encapsulation of bioactive compounds, etc. [7]. It should be noted that the formation of nanostructures requires a sufficient understanding of the process and factors affecting it [8]. It has also been demonstrated that the synthesis of CSNPs using the ionic gelation method possesses many advantages compared to conventional methods such as micelle formation [9], coacervation [10], spray drying [11], emulsification [12], and solvent evaporation [13].

Ionic gelation is a simple, controllable, and convenient technique for the fabrication of CSNPs, without any need for organic solvents and/or toxic chemicals [14,15,16]; apart from that, the size and zeta potential (ZP) of CSNPs can be easily controlled by adjusting the concentration of CS and sodium tripolyphosphate (STPP), CS to STPP ratio, pH, etc. [17]. It should be noted that increasing the ratio of CS to STPP indicates an increase in the concentration of CS and/or a decrease in the concentration of STPP, simultaneously. In addition, CS is used in ionic interactions due to its cationic nature (pK_a_ = 6.5) and the presence of free hydroxyl and amine groups [17,18]. It results in adequate interactions with negatively charged species such as sodium sulfate and STPP (Na_5_P_3_O_10_) [19]. CS nano/microparticles have already been produced through chemical cross-linking with glyoxal, ethylene glycol diglycidyl ether, and glutaraldehyde [18,20]. However, the latter showed physiological toxicity; therefore, their application in the food and drug industries is limited [21].

When preparing CSNPs using the ionic gelation method, a cross-linking agent such as STPP is used to decrease the mobility and increase the stability of the CSNPs [22]. It has been revealed that STPP releases phosphoric and hydroxyl ions by dissolving in water along with polyanions (P_3_O_10_^5−^), which build the inter- and intramolecular linkages with the NH_2_^+^ groups of CS [19]. It should also be noted that the phosphoric ion mentioned is distinct from the tripolyphosphate (TPP) ion formed upon the dissolution of STPP. Apart from its multivalent and nontoxic characteristics, STPP is can interact with CS under mild conditions such as ambient temperatures and pH values [3,23].

Considering that one of the important applications of CSNPs is in the field of nutraceutical and drug delivery, a wide spectrum of ingredients—regardless of whether they are hydrophilic, hydrophobic, or bacterial—can be encapsulated using CS-based systems [7]. For example, chlorogenic acid was encapsulated within CSNPs with a regular distribution and size of around 210 nm [24]. In another study [25], tuberose fragrance was encapsulated using CSNPs (TC-NPs) with 1.5 mg/mL of CS and the weight ratio of CS:STPP = 5:1. They reported a particle size, polydispersity index (PDI), and ZP of the TC-NPs about 174 nm, 0.14, and 20.8 mV, respectively. Similarly, risperidone [15], selenite [26], L-ascorbic acid [27], ascorbyl palmitate [28], tea polyphenols [29], catechin [30,31], quercetin [32], rutin [33], carvacrol [34], and clove essential oil [35] have been encapsulated within CSNPs. Recent studies have also revealed that CSNPs could be applied to stabilize the oil-in-water (O/W) Pickering emulsions, which presents excellent advantages such as high stability, good mechanical properties, reduced foaming problems, and less toxicity than conventional emulsions [36,37,38]. Briefly, Pickering emulsions stabilized with CSNPs can be used to encapsulate and carry the nutraceuticals to control lipid digestibility, retard the oxidation of lipids in food formulations, enhance the properties of edible films, and incorporate them in complex food systems [39]. It should be noted that the stability of Pickering emulsions directly depends on different factors such as oil/water ratio, shape and concentration of NPs, surface activity, pH, and ionic strength [40]. As an example, CSNPs were synthesized by preparing a 0.1% (*w*/*v*) CS solution, and adjusting the pH value [41]; the obtained CSNPs were used as emulsifiers to produce Pickering emulsions.

In addition, it is suggested that some properties of synthesized CSNPs, including their cross-linking density, hydrophilicity, and crystallinity, are critical for special applications, e.g., the controlled release of bioactive compounds [18,19]. It has also been shown that the technical parameters, such as CS molecular weight, and CSNPs synthesis technique considerably affect the functionality of CSNPs. For example, low molecular weight (LMW) CS presents higher biocompatibility, biodegradability, bioactivity, solubility, and less toxicity compared with high molecular weight (HMW) CS [17]. Furthermore, it has been reported that once the molecular weight and CS concentration increase, larger particles are formed. When applying LMW CS, it is easier to control the size of particles and their distribution due to the decrease in the entanglement of the CS chains and the lower viscosity of the internal aqueous phase [42,43,44].

However, the optimal formation of CSNPs with high stability and mono-dispersity remains the main challenge, particularly where the application of Pickering emulsions stabilized by CSNPs is required on an industrial scale (i.e., nutraceutical and drug delivery systems). It has been shown that applying CSNPs with small sizes can significantly reduce the emulsion stability over time [39]. In this regard, many variables dramatically affect the size, morphology, and other properties of CSNPs during the ionic gelation fabrication method, where STPP—as the most common cross-linking agent—is used. The optimization process of CSNPs using a simple but efficient method (i.e., ionic gelation) could also significantly facilitate and improve the encapsulation of bioactive compounds without using syntactic and/or expensive emulsifiers. To the best of our knowledge, there is not a comprehensive reference that particularly investigates CSNPs modeling and optimization, as well as evaluating the most important properties of the CS-TPP system so that the data provided could be used practically to design and develop such systems; it should be mentioned that the current literature has only been focused on one or selective properties of CSNPs which considerably limits their results in terms of developing complex emulsion systems [17,22,45].

Accordingly, this study aimed to design and optimize reproducible CSNPs with optimized size distribution and sufficient stability, as well as to investigate a wide range of properties of the CS-STPP system—using sophisticated tools—that can be used as appropriate stabilizers for bioactive compound-loaded Pickering emulsions. Therefore, in this work, we particularly studied the effect of manipulating selected parameters including the different concentrations of LMW CS (1, 3, and 5 mg/mL) and STPP (0.5, 0.75, and 1 mg/mL), as well as various CS to STPP ratios (1:1, 3:1, and 5:1) on the physicochemical properties of CSNPs. We also optimized the production process using response surface methodology (RSM) as a combined statistical and mathematical route utilized for the optimization, modeling, and interpretation of the effect of system variables on the desirable targets with the aim of producing the desirable particles and stable emulsion by knowing the effects of relevant parameters on the CSNPs properties. A second-order polynomial equation was also introduced to predicate the particle size, PDI, and ZP. In addition, the structural and morphological properties of CSNPs, interactions between CS and STPP, as well as their thermal behaviors and crystallinity were characterized using field emission scanning electron microscopy (FE-SEM), attenuated total reflection Fourier-transform infrared (ATR-FTIR) spectroscopy, DSC, TGA, and XRD techniques.

## 2. Materials and Methods

### 2.1. Materials

CS (LMW = 50–190 kDa and degree of deacetylation between 75–85%) was purchased from Sigma-Aldrich, St. Louis, MO, USA. STPP, acetic acid, and sodium hydroxide were also supplied by Merck, Darmstadt, Germany. All other chemicals used were of analytical grade.

### 2.2. Production of Chitosan Nanoparticles

CSNPs were synthesized through the ionic gelation technique as previously described [46], with some modifications. Different concentrations of CS (1, 3, and 5 mg/mL) were dissolved in an aqueous solution of acetic acid (1% *v*/*v*). Then, the pH of the CS solution was adjusted in the range of 4.7 to 4.8 using 2 M sodium hydroxide. STPP (0.5, 0.75, and 1.0 mg/mL) was also dissolved in deionized water and the pH value was adjusted to 4 using acetic acid. In order to remove impurities and undissolved particles of CS and STPP, their solutions were filtered through 0.45 and 0.22 µm syringe filters, respectively. Finally, the STPP solution was added dropwise to the CS solution at room temperature, while stirring the CS solution at 800 rpm with a magnetic stirrer until an opalescent suspension was obtained. The formation of CSNPs automatically began through the initiation of the ionic gelation mechanism induced by STPP.

### 2.3. Characterization of Chitosan Nanoparticles

The average particle size, PDI, and ZP of the CSNPs were determined using a Zetasizer (MAL1001767, Malvern Instruments, Malvern, UK) at 25 °C. For the morphological analysis, CSNPs were coated with a thin gold–palladium layer by a sputter coater unit (Coater Q150T, Quorum Technologies, East Sussex, UK), and their microstructure was evaluated using FE-SEM (MIRA3, Tescan, Brno, Czech Republic). The average diameter was also determined using ImageJ software (Version 1.46, National Institutes of Health, Bethesda, MD, USA) from a minimum of 100 random measurements [19]. ATR-FTIR spectra of the CS, STPP, and CSNPs were obtained with an ATR-FTIR spectrometer (Tensor II, Bruker, spectrometer, Billerica, MA, USA) to characterize the chemical structure of CSNPs. The spectra were scanned over the wave number range of 500 to 4000 cm^−1^, and the spectral data were processed using an OMNIC software package (version 9.2.86, Thermo Fisher Scientific Inc., Waltham, MA, USA) [38].

The thermal properties of CS, STPP, and CSNPs were analyzed using a differential scanning calorimetry (DSC) instrument (DSC 200 F3, Netzsch, Selb, Germany), at a constant rate of 10 °C/min, over a temperature range of 25 to 300 °C. Indium and silver standards were used to calibrate the DSC enthalpy and temperature scale. Furthermore, an empty aluminum container and nitrogen gas were applied as the reference and atmosphere, respectively [24]. The thermal degradation behavior of samples was also determined by TGA (Q600, TA Instruments, New Castle, DE, USA) by heating the samples from 25 to 625 °C at a heating rate of 15 °C/min in an argon atmosphere [38]. For the crystallinity analysis of CSNPs, the X-ray diffraction (XRD) patterns of CS, STPP, and CSNPs were recorded on an X-ray diffractometer (PW 1730, Philips, Amsterdam, The Netherlands). The samples were irradiated using monochromatized Cu Kα radiation (1.54056 °A) and analyzed between 7 and 80° (2θ). The current, voltage, step size, and time per step were 30 mA, 40 kV, 0.05°, and 1 s, respectively [19].

### 2.4. Statistical Analysis

The RSM is a combined statistical and mathematical route utilized for optimization, modeling, and interpretation of the effect of system variables on desirable targets [47]. To investigate the effect of different factors on the properties of CSNPs, the experimental design was performed and analyzed using RSM—central composite design (CCD)—with 20 runs, and six replications at the central point through analysis of variance (ANOVA) for a statistical significance *p* < 0.05, by Design Expert software (version 11, State-Ease Co., Minneapolis, MN, USA). Three independent variables (CS concentration, STPP concentration, and CS to STPP ratio) with three levels were investigated (Table 1).

It should be noted that the range of these variables was determined according to pre-tests, as well as previous studies. For each point, the size, ZP, and PDI of the CSNPs were also considered as the responses. The main target of our work was to optimize the production conditions for medium-sized CSNPs with an acceptable PDI and ZP. A second-order polynomial equation was also applied to determine the particle size, PDI, and ZP (Equation (1)).

(1) $$Y=a_{0}+a_{1}X_{1}+a_{2}X_{2}+a_{3}X_{3}+a_{11}X_{1}^{2}+a_{22}X_{2}^{2}+a_{33}X_{3}^{2}+a_{12}X_{1}X_{2}+a_{13}X_{1}X_{3}+a_{23}X_{2}X_{3}$$

where $a_{0}$ is a constant and $a_{i}$, $a_{ii}$, and $a_{ij}$ are also the linear, quadratic, and interactive coefficients, and $X_{1}$, $X_{2}$, and $X_{3}$ are the CS concentration, STPP concentration, and CS/STPP ratio, respectively.

## 3. Results and Discussion

### 3.1. Changes in the Size and Zeta Potential of Chitosan Nanoparticles

It has been reported that CSNPs can be formed in special concentrations of CS and STPP [22]. Moreover, adding STPP into acidic aqueous CS with various ratios results in the formation of nano/macro-suspensions, and/or sedimentation [15]. If the STPP volume is very low and inadequate, it cannot induce the cross-linking of CS and the resulting mixture would be a clear solution. In one study [22] 5 mL of STPP solution (with different concentrations) was added to 10 mL of a 0.5 mg/mL CS solution. They revealed that when the STPP level was very low (i.e., <1.5 mg), the reaction solution was clear like pure CS solution. Furthermore, the pH value of CS and STPP solutions affect the physicochemical attributes of CSNPs; CS in a pH below its pK_a_ is charged positively because of the protonation of its amino groups; at a pH > 6.5, larger aggregates are formed due to the deprotonation of CS [36]. On the other hand, STPP ions are multivalent, with their valences ranging between −2 to −5 depending on the pH level, which affects the ionic species and charge number of STPP [48]. For these reasons, in this study, the different concentrations and ratios of CS to STPP were used to fabricate CSNPs with uniform size, low PDI, and a high ZP.

**Figure 1:**
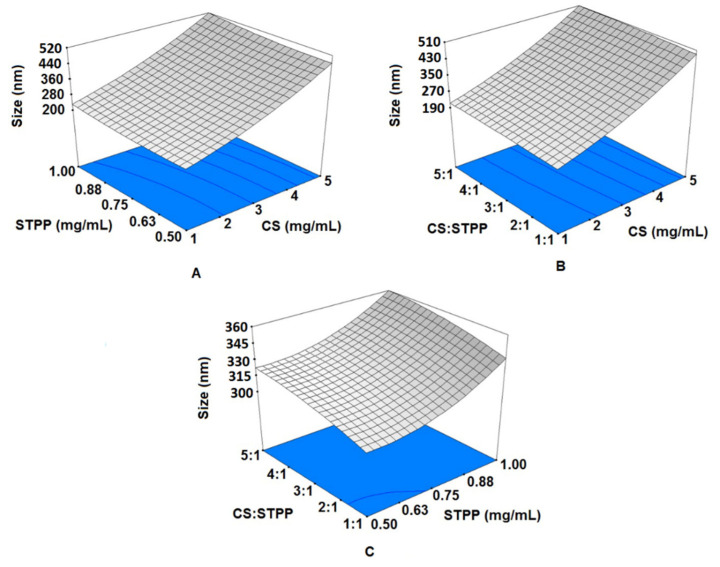
The influence of (**A**) chitosan (CS) and sodium tripolyphosphate (STPP) levels, (**B**) CS levels and the ratio of CS:STPP, and (**C**) STPP levels and the ratio of CS:STPP on the size of produced CS nanoparticles (CSNPs).

The size of prepared CSNPs was measured from 190 to 520 nm, as shown in Figure 1. It has been reported that the size of CSNPs could be <1000 nm, in general [49]. It was revealed that the size of CSNPs had a linear relationship with the concentration of CS and STPP (Figure 1A); the higher the concentrations, the bigger the size of CSNPs. This finding is in agreement with the previous works [17,24]. This phenomenon can be attributed to the stronger intramolecular repulsions most probably due to the high level of the non-neutralized –NH_3_^+^ which consequently results in higher CS concentrations causing the CS chain stretching and larger CSNPs [31]. It was also proven that a higher viscosity of a gelation medium with a higher CS concentration can lead to an increase in liquid phase resistance against dispersion and a larger size of CSNPs [15]. It seems that the addition of STPP not only increases the ionic strength but also increases the possibility of particles being bridged by their ions into macroscopic aggregates. When the ratio of CS to STPP increased (Figure 1B,C), the particle size also increased, similar to the results of other studies [19,42]; nonetheless, a reverse trend has also been observed [31]. A uniform and narrow distribution for the size of CSNPs have been reported and the average CSNPs size decreased from 522.2 to 213.8 nm while the CS:STPP mass ratio changed from 5:2 to 5:5 [3]. It has been stated that [17] at LMWs of CS < 2.0 mg/mL, the intermolecular electrostatic repulsion and the intermolecular hydrogen bonding attraction are in balance, and by increasing the CS level, CS molecules are becoming closer together; thus, intermolecular the cross-linking rate is increased and so the particle size gets bigger. It can also be explained that when there is a higher STPP content compared to NH_3_^+^ groups, larger particles are formed due to the cross-linking of multiple smaller mono particles. In other words, once the STPP amount increases, CS molecules are completely cross-linked, and the remaining STPP can cause more CS molecules to get involved in the formation of a single CSNP and hence the subsequent larger particle size [17,22]. In contrast, when the STPP level is low, limited cross-linking among particles is initiated.

The PDI describes the distribution of particles with a range between 0 and 1 [50]. It has been stated that a PDI value > 0.5 shows the polydisperse distribution, which is why it is desirable to maintain the PDI as low as possible [19,51]. In addition, the main factor controlling dispersion stability is the ZP [14]. It has been reported that once the ZP is >+30 mV and <−30 mV, a suspension presents higher physical stability [24]. On the other hand, in order to avoid aggregation, it is necessary to have a high positive charge in CSNPs [19]. In this study, CSNPs had a PDI in a range between 0.18 and 0.48 which indicated a uniform and appropriate distribution of CSNPs. Similarly, the particle size and PDI increased with increasing CS concentration, from 125 to 304 nm and from 0.16 to 0.28, respectively [26].

The results of this study showed that the ZP of CSNPs was positive and increased with increasing CS concentration and CS/STPP ratio, most probably due to the increase in the amino groups [19]. The ZP decreased at higher STPP concentrations, presumably as a result of a greater neutralization of CS amino groups by STPP [17,22]. Similarly, the size of CSNPs reduced from 814 to 322 nm for the 2:1 to 5:1 CS:STPP mass ratios, whereas the ZP of particles increased from +21 to +59 mV since the STPP had a limited capacity to neutralize protonated amino groups in the CS [33].

Furthermore, ANOVA data (Table 2) revealed that the quadratic models appropriately represented the experimental data for particle size, PDI, and ZP with high coefficients of determinations (*R*^2^) of 0.9915, 0.9964, and 0.9735, respectively. All of the independent variables had a significant effect (*p* < 0.05) on the particle size, PDI, and ZP. The quadratic term of CS concentration was significant (*p* < 0.01) for particle size and the PDI. The quadratic term for the STPP concentration had a significant effect on the ZP; moreover, the interactive term of $a_{13}$ was significant for the PDI and ZP (*p* < 0.05).

Finally, according to ANOVA, predicated models for the effect of CS concentration, STPP concentration, and CS/STPP ratio on the particle size, PDI, and ZP of CSNPs were calculated according to Equations (2)–(4).

(2) $$Particle\;size=322.96+142.9X_{1}+17.4X_{2}+9.9X_{3}+26.59X_{1}^{2}$$

(3) $$PDI=0.37+0.12X_{1}+0.023X_{2}+0.013X_{3}-0.042X_{1}^{2}-\left(6.75\times 10^{-3}\right)X_{1}X_{3}$$

(4) $$ZP=35.21+11.86X_{1}-4.19X_{2}+5.31X_{3}-3.31X_{2}^{2}-2.34X_{1}X_{3}$$

**Figure 2:**
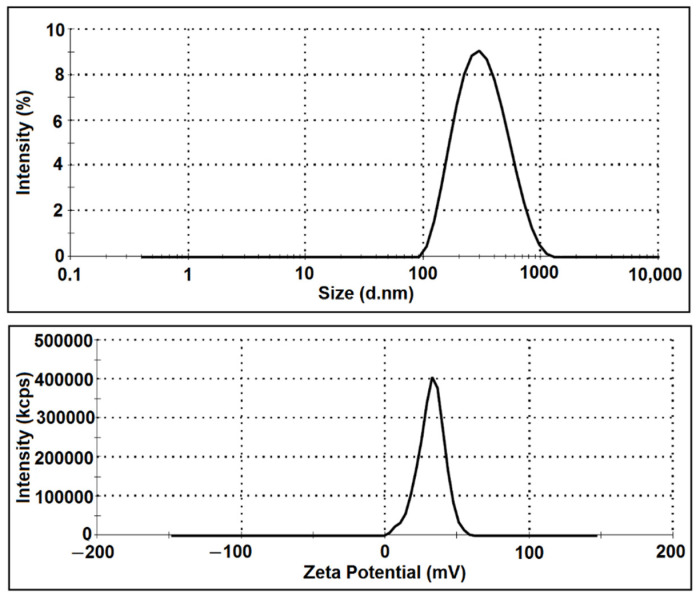
The Particle size and zeta potential (ZP) distribution of produced chitosan nanoparticles (CSNPs) by using 3 mg/mL of CS, 1 mg/mL of STPP, and a CS to STPP ratio of 3:1.

According to the results of our study, by using 3 mg/mL of CS, 1 mg/mL of STPP, and a CS to STPP ratio of 3:1, medium-sized NPs with an acceptable PDI and ZP were obtained (Figure 2). The agreement between the experimental data and the predicted values also confirmed the predictability of the RSM model resulting from the optimized conditions (Table 3).

### 3.2. Morphology of Chitosan Nanoparticles

**Figure 3:**
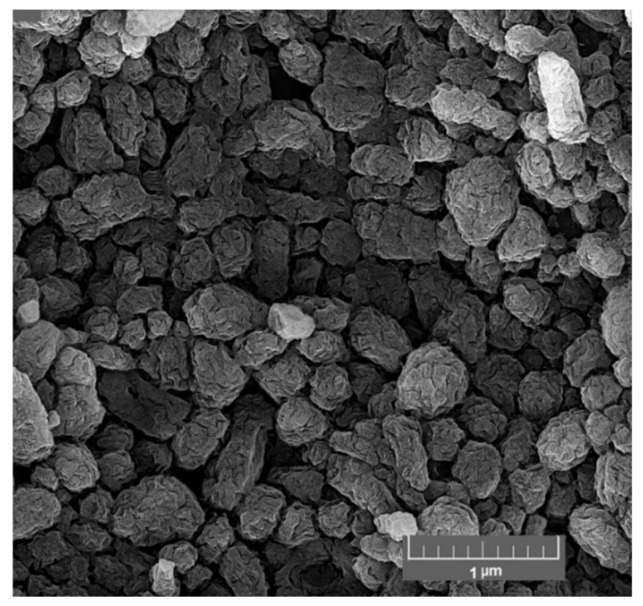
FE-SEM image of chitosan nanoparticles (CSNPs). (3 mg/mL of CS, 1 mg/mL of STPP, and a CS to STPP ratio of 3:1).

According to FE-SEM images, the CSNPs showed a rough surface and a diameter in the range of 168 to 485 nm (average diameter of 262 nm) (Figure 3), similar to the results another study [52]. The different sizes of CSNPs measured by dynamic light scattering (DLS), Section 3.1, and FE-SEM, could be attributed to the swelling of CSNPs in aqueous media and measuring the hydrodynamic diameter in DLS; whereas the actual diameter of particles in dried form was determined using FE-SEM [14,17]. On the other hand, since the hydrogen bonding interactions between CSNPs prevail gently during the drying process, some aggregation occurs in the nanoparticles. The well-established ionic bridging of the particles—resulting from the surface-bound TPP—can also be attributed to the aforementioned phenomena.

### 3.3. Chemical Interactions in Chitosan Nanoparticles

**Figure 4:**
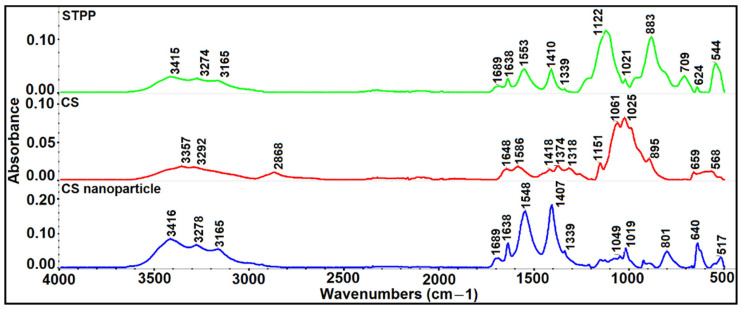
ATR-FTIR spectra of chitosan (CS), sodium tripolyphosphate (STPP), and CS nanoparticles (CSNPs).

The ATR-FTIR spectra of CS, STPP, and CSNPs are illustrated in Figure 4. The appearance, disappearance, and/or shift of bands can reveal the interactions between CS and STPP [36]. The broad absorption bands at 3357 and 3292 cm^−1^ indicate the NH_2_ and OH groups stretching in CS. Further, the bands at 2868, 1648, and 1586 cm^−1^ correspond to the C–H stretching vibrations, C=O stretching from amide I, as well as N–H bending and C–N stretching from amide II, respectively. Furthermore, the bonds assigned to CH_2_ bending, CH_3_ symmetrical deformation, and primary/secondary OH in plane bending in the ATR-FTIR spectra of CS appeared at 1418, 1374, and 1318 cm^−1^, respectively. The other prominent bands of CS were observed at 1061 cm^−1^ (amine C–N stretching) and 1025 cm^−1^ (skeletal vibration of C–O stretching). These results were in excellent agreement with those reported by two previous works [3,36].

The ATR-FTIR spectra also indicated that the bands at 1122 and 883 cm^−1^ relate to the bending and extending of the vibration of P=O in the STPP, similar to the results of other studies [14,19]. In CSNPs (Figure 4), the absorption band that appears at 3416 cm^−1^ (stretching of O–H) and shifting bands to 3278 and 3165 cm^−1^ indicate the presence of the stretching vibration of the NH_2_ and OH groups. It should be noted that the peak intensity of the OH band at 3000–3500 cm^−1^ increased, most probably due to the enhancement of hydrogen bonding. In addition, due to interactions between CS and STPP, the bands of amide I and amide II dramatically shifted to 1638 and 1548 cm^−1^ and the P=O groups, which were also observed in the absorption bands at wavenumbers 1049 and 1019 cm^−1^. It seems that increasing the peak intensity of the angular deformation of the CH_2_ group at 1407 cm^−1^ can be attributed to rising the polarization of the carbonyl group upon its interaction [50,53,54]. The formation of this group confirmed the interaction between phosphate groups of STPP and CS by ionic bonds. In other words, the presence of functional groups of CS and STPP in CSNPs can clearly prove the formation of CSNPs. Moreover, some studies have demonstrated that the addition of STPP might improve the mechanical properties of CS most probably due to increasing the ionic cross-linking [50,55].

### 3.4. Thermal Properties of Chitosan Nanoparticles

**Figure 5:**
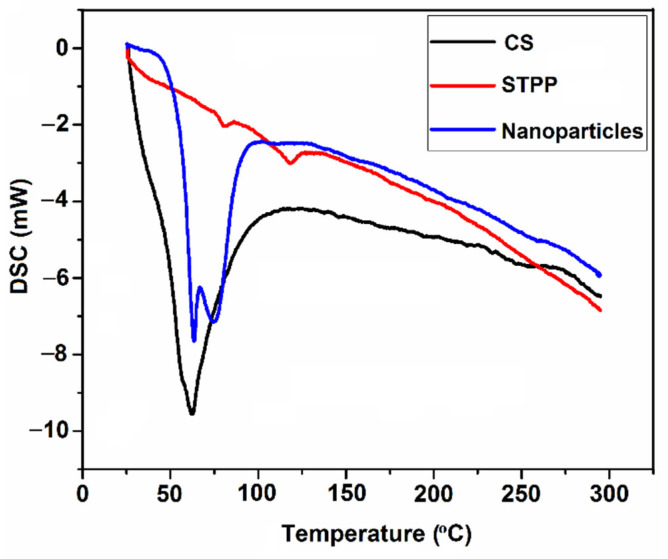
DSC thermograms of chitosan (CS), sodium tripolyphosphate (STPP), and CS nanoparticles (CSNPs).

Macromolecules, such as polysaccharides, generally have a high affinity with water; therefore, they may be hydrated in a solid state due to having primary and supramolecular structures [18,30]. Hence, the molecular changes after ionic interaction between CS and STPP can be explained by studying the endotherms related to the evaporation of water in the samples [18]. The DSC thermograms of CS, STPP, and CSNPs are shown in Figure 5.

The CS presented an endothermic peak at 62.4 °C (ΔH = 131.30 J/g) which can most probably be attributed to the evaporation and elimination of absorbed and bound water. This phenomenon may also demonstrate the high affinity of water with CS [56,57,58]. However, it has been reported that CS has endothermic and exothermic peaks at 100 °C and 306 °C, respectively [19]. They also claimed that this exothermic peak may indicate degradation most probably due to depolymerization and dehydration. The pure STPP showed an endothermic peak at 118.2 °C (ΔH = 4.15 J/g), corresponding to the melting point; however, the aforementioned endothermic peak was not observed in the CSNPs, most probably due to the ionic interaction between STPP and CS [19]. Instead, the CSNPs resulted in a double sharp endothermic peak at about 74.5 °C (ΔH = 122.00 J/g), which could be ascribed to the structural change process during heating near the melting temperature. It seems that the differences in position and area of the first endothermic peak can probably be attributed to the various water-holding capacities and interaction strengths between water and polymers [57]. The authors in a previous study [30] observed two endothermic peaks associated with CS and CSNPs so that the first peak was in the onset and end between 110.80 and 124.70 °C.; also it has been revealed that the CS and CSNPs have exothermic peaks at 324.30 °C (ΔH = −23.80 J/g) and 259.60 °C (ΔH = −79.80 J/g), respectively [57]. The shift of endothermic peaks for structures containing CS is related to the presence of hydrophobic and hydrophilic groups that can decrease and/or increase the binding of CS to water molecules [56]. It should be noted that some differences in glass transition and melting temperatures of CS and CSNPs in this study—compared with the previous reports—can be ascribed to the variety of CS molecular weights, methodologies of particle preparation, as well as to the degree of deacetylation, which dramatically affects the physicochemical properties of samples.

### 3.5. Thermal Stability and Degradation of Chitosan Nanoparticles

**Figure 6:**
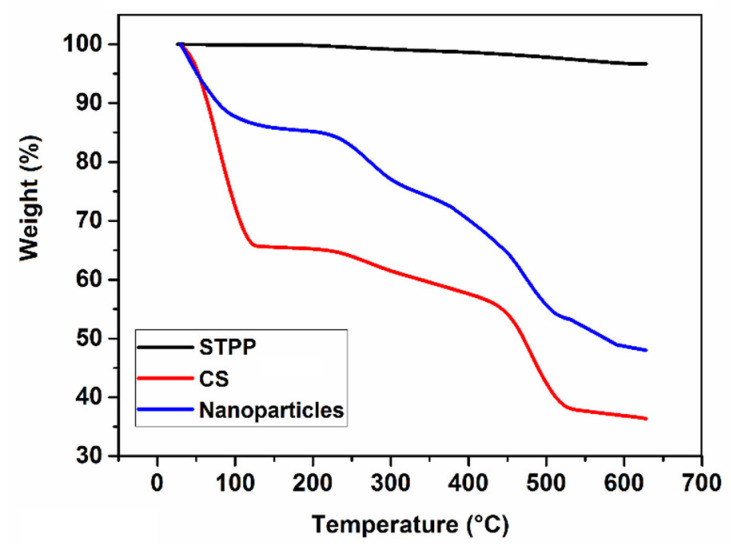
TGA thermograms of chitosan (CS), sodium tripolyphosphate (STPP), and CS nanoparticles (CSNPs).

TGA is a method for investigating the thermal stability and weight changes in samples [16,34]. According to the TGA thermograms (Figure 6), STPP, CS, and CSNPs exhibited weight losses of approximately 3.30%, 63.60%, and 52.00%, respectively. The initial weight losses of CS and CSNPs, observed in the range of 25 to 150 °C, demonstrated the evaporation of absorbed water [16]; the weight losses shown in the temperature range of 150 to 600 °C could be attributed to the decomposition and degradation of the polymer chain of the free CS and CS cross-linked with STPP, the breakage of hydrogen bonds between the N-acetyl and free amino groups, the gradual oxidative degradation of carbonaceous residue, and byproducts formed during the previous steps [59,60].

The STPP, CS, and CSNPs exhibited the first degradation step at 427.10, 85.50, and 40.70 °C, respectively. In general, CSNPs have higher thermal stability than CS due to the presence of phosphate groups in STPP. In a similar work [61], it was also revealed that CS and CSNPs had maximum weight losses of about 70% and 45% at 800 °C, respectively.

### 3.6. Crystallinity of Chitosan Nanoparticles

**Figure 7:**
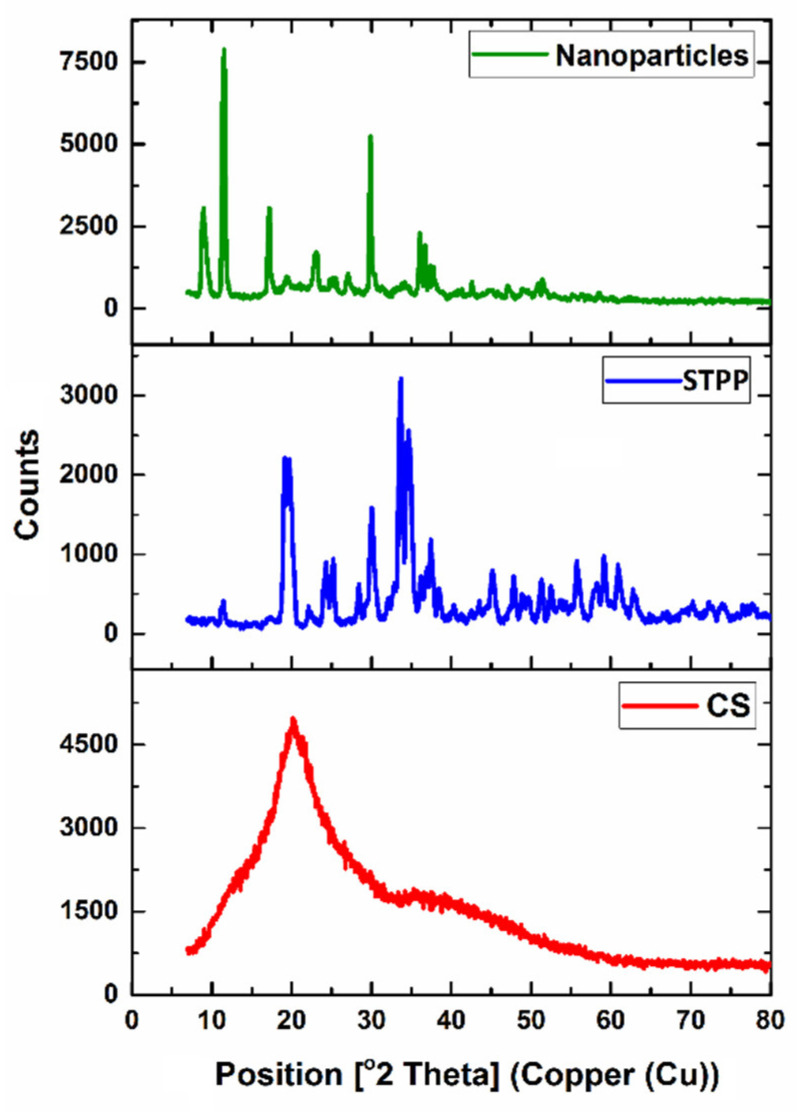
The XRD patterns of chitosan (CS), sodium tripolyphosphate (STPP), and CS nanoparticles (CSNPs).

The materials can have amorphous, crystalline, and/or both structures. It has been reported that amorphous structures have a generally higher and faster solubility than crystalline structures [62]. In the XRD pattern, it has been demonstrated that the broadened peaks result from imperfect crystals, and the width of the peak is related to the size of the crystallite [63]. The XRD patterns of CS, STPP, and CSNPs are illustrated in Figure 7. The CS gives a characteristic crystalline peak at 2θ = 20.26° with an approximately semi-crystalline structure, similar to that in other works [54,64]. Whereas, the XRD diffractogram of STPP showed multiple strong peaks between 11.3° and 74°, the most evident peaks were observed at 2θ = 19.05°, 19.77°, 33.60°, and 34.49°. We found many differences in the XRD pattern of CS and CSNPs, in which the peak at 2θ = 20.26° in CS disappeared and shifted to the lower values; moreover, the new strong peaks at 2θ = 9.01°, 11.39°, 11.61°, 17.16°, and 29.83° were formed after the ionic interaction with STPP, showing the change in the CS packing structure. The results also revealed a greater chain alignment in the CSNPs due to cross-linking between CS and STPP. Conversely, it has been reported [57] that the CS and CSNPs have crystalline and amorphous structures, respectively. It was also reported that CSNPs show a higher intensity peak at 2θ = 19.85° than CS [54]. Generally, shifting the peaks and increasing the peak intensity in CSNPs could be attributed to the ionic interaction between CS and STPP; thus, the CS semi-crystalline structure was converted to a structure with a high degree of crystallinity in the CSNPs; these results also prove the formation of CSNPs.

## 4. Conclusions

CSNPs, as nanostructures with unique attributes, can be fabricated through electrostatic interactions between CS and STPP with many potential applications in the food and drug industries. In this study, CSNPs were formed by applying the ionic gelation method and using STPP as a cross-linking agent. Due to the interactions between amino groups in the CS solution and the P_3_O_10_^5−^ groups in STPP, in CSNPs, the bands of amide I and amide II dramatically shifted to 1638 and 1548 cm^−1^ and the P=O groups were also observed in the absorption bands at wavenumbers of 1049 and 1019 cm^−1^. Our results showed that the size of CSNPs increased at higher concentrations of CS, STPP, and higher ratios of CS to STPP; moreover, the ZP of CSNPs was positive and increased with increasing CS concentration and CS/STPP ratio due to the increase in the amine groups. Furthermore, ANOVA results revealed that the quadratic models appropriately represented the experimental data for particle size, PDI, and ZP with high coefficients of determinations (*R*^2^) of 0.9915, 0.9964, and 0.9735, respectively. The results showed that by using 3 mg/mL of CS, 1 mg/mL of STPP, and a CS to STPP ratio of 3:1, medium-sized NPs with an acceptable PDI and ZP were produced. According to FE-SEM images, the CSNPs presented an average size of 262 nm and a rough surface. The CS had an endothermic peak at 62.4 °C (ΔH = 131.30 J/g) that can be probably associated with the evaporation and elimination of absorbed and bound water, whereas, the CSNPs showed a double sharp endothermic peak at about 74.5 °C (ΔH = 122.00 J/g). The STPP, CS, and CSNPs exhibited the first degradation step at 427.10, 85.50, and 40.70 °C, respectively. Generally, the CSNPs had higher thermal stability than CS due to the presence of phosphate groups in the STPP. Ionic interactions between CS and STPP were confirmed by XRD patterns and the CS semi-crystalline structure was converted to a structure with a high degree of crystallinity within the CSNPs. Finally, the optimized CSNPs in this work were found to be good candidates for use as a food-grade carrier to encapsulate and deliver bioactive compounds as well as appropriate materials to properly stabilize Pickering emulsions.

## Figures and Tables

**Table 1:** The experimental design to produce the chitosan nanoparticles (CSNPs).

Factor	Levels
Chitosan (CS) concentration (mg/mL)	1, 3, and 5
Sodium tripolyphosphate (STPP) concentration (mg/mL)	0.5, 0.75, and 1
CS:STPP ratio	1:1, 3:1, and 5:1

**Table 2:** Analysis of variance (ANOVA) for the coefficients of the quadratic equation.

	Particle Size		PDI		ZP	
Variable	Coefficients	*p* Value	Coefficients	*p* Value	Coefficients	*p* Value
$a_{0}$	322.96	<0.0001	0.37	<0.0001	35.21	<0.0001
$a_{1}$	142.90	<0.0001	0.12	<0.0001	11.86	<0.0001
$a_{2}$	17.40	0.0023	0.023	<0.0001	−4.19	0.0003
$a_{3}$	9.90	0.0432	0.013	0.0002	5.31	<0.0001
$a_{11}$	26.59	0.0086	−0.042	<0.0001	−1.36	0.3688
$a_{22}$	10.09	0.2444	3 × 10^−3^	0.5146	−3.31	0.0452
$a_{33}$	−3.41	0.6849	3.5 × 10^−3^	0.4488	−2.61	0.1014
$a_{12}$	1.38	0.7796	1.5 × 10^−3^	0.5772	−0.038	0.9657
$a_{13}$	−1.12	0.8188	−6.75 × 10^−3^	0.0268	−2.34	0.0204
$a_{23}$	−0.37	0.9391	−5 × 10^−4^	0.8515	−1.14	0.2102

**Table 3:** Experimental validation data of the optimal conditions.

	Experimental	Predicted
Size (nm)	359	355
PDI	0.32	0.35
ZP (mV)	30.2	32.5

## Data Availability

The data supporting the results of this study are included in the present article.

## References

[1] Vozza G., Khalid M., Byrne H.J., Ryan S.M., Frias J.M. (2019). Nutraceutical formulation, characterisation, and in-vitro evaluation of methylselenocysteine and selenocystine using food derived chitosan:zein nanoparticles. Food Res. Int..

[2] Yang Y., Fang Z., Chen X., Zhang W., Xie Y., Chen Y., Liu Z., Yuan W. (2017). An Overview of Pickering Emulsions: Solid-Particle Materials, Classification, Morphology, and Applications. Front. Pharmacol..

[3] Shah B.R., Li Y., Jin W., An Y., He L., Li Z., Xu W., Li B. (2016). Preparation and optimization of Pickering emulsion stabilized by chitosan-tripolyphosphate nanoparticles for curcumin encapsulation. Mater. Sci. Eng. C.

[4] Hosseinnejad M., Jafari S.M. (2016). Evaluation of different factors affecting antimicrobial properties of chitosan. Int. J. Biol. Macromol..

[5] Maleki G., Milani J.M., Gopi S., Thomas S., Pius A. (2020). Chapter 6—Functional properties of chitin and chitosan-based polymer materials. Handbook of Chitin and Chitosan.

[6] Akbari-Alavijeh S., Shaddel R., Jafari S.M. (2020). Encapsulation of food bioactives and nutraceuticals by various chitosan-based nanocarriers. Food Hydrocoll..

[7] Divya K., Jisha M.S. (2018). Chitosan nanoparticles preparation and applications. Environ. Chem. Lett..

[8] Augustin M.A., Sanguansri P. (2009). Chapter 5—Nanostructured Materials in the Food Industry. Advances in Food and Nutrition Research.

[9] Zhang C., Ding Y., Ping Q., Yu L. (2006). Novel chitosan-derived nanomaterials and their micelle-forming properties. J. Agric. Food Chem..

[10] Baruch L., Machluf M. (2006). Alginate–chitosan complex coacervation for cell encapsulation: Effect on mechanical properties and on long-term viability. Biopolym. Orig. Res. Biomol..

[11] Asada M., Takahashi H., Okamoto H., Tanino H., Danjo K. (2004). Theophylline particle design using chitosan by the spray drying. Int. J. Pharm..

[12] Ribeiro A.J., Silva C., Ferreira D., Veiga F. (2005). Chitosan-reinforced alginate microspheres obtained through the emulsification/internal gelation technique. Eur. J. Pharm. Sci..

[13] Lee M., Cho Y.W., Park J.H., Chung H., Jeong S.Y., Choi K., Moon D.H., Kim S.Y., Kim I.-S., Kwon I.C. (2006). Size control of self-assembled nanoparticles by an emulsion/solvent evaporation method. Colloid Polym. Sci..

[14] Wu J., Wang Y., Yang H., Liu X., Lu Z. (2017). Preparation and biological activity studies of resveratrol loaded ionically cross-linked chitosan-TPP nanoparticles. Carbohydr. Polym..

[15] Mudhakir D., Wibisono C., Rachmawati H. (2014). Encapsulation of risperidone into chitosan-based nanocarrier via ionic binding interaction. Procedia Chem..

[16] Hosseini S.F., Zandi M., Rezaei M., Farahmandghavi F. (2013). Two-step method for encapsulation of oregano essential oil in chitosan nanoparticles: Preparation, characterization and in vitro release study. Carbohydr. Polym..

[17] Fan W., Yan W., Xu Z., Ni H. (2012). Formation mechanism of monodisperse, low molecular weight chitosan nanoparticles by ionic gelation technique. Colloids Surf. B Biointerfaces.

[18] Bhumkar D.R., Pokharkar V.B. (2006). Studies on effect of pH on cross-linking of chitosan with sodium tripolyphosphate: A technical note. AAPS PharmSciTech.

[19] Nair R.S., Morris A., Billa N., Leong C.-O. (2019). An evaluation of curcumin-encapsulated chitosan nanoparticles for transdermal delivery. AAPS PharmSciTech.

[20] Gupta K.C., Jabrail F.H. (2006). Glutaraldehyde and glyoxal cross-linked chitosan microspheres for controlled delivery of centchroman. Carbohydr. Res..

[21] Berger J., Reist M., Mayer J.M., Felt O., Peppas N., Gurny R. (2004). Structure and interactions in covalently and ionically crosslinked chitosan hydrogels for biomedical applications. Eur. J. Pharm. Biopharm..

[22] Al-Nemrawi N., Alsharif S., Dave R. (2018). Preparation of chitosan-TPP nanoparticles: The influence of chitosan polymeric properties and formulation variables. Int. J. Appl. Pharm..

[23] Zhao L.-M., Shi L.-E., Zhang Z.-L., Chen J.-M., Shi D.-D., Yang J., Tang Z.-X. (2011). Preparation and application of chitosan nanoparticles and nanofibers. Braz. J. Chem. Eng..

[24] Nallamuthu I., Devi A., Khanum F. (2015). Chlorogenic acid loaded chitosan nanoparticles with sustained release property, retained antioxidant activity and enhanced bioavailability. Asian J. Pharm. Sci..

[25] Xiao Z., Tian T., Hu J., Wang M., Zhou R. (2014). Preparation and characterization of chitosan nanoparticles as the delivery system for tuberose fragrance. Flavour Fragr. J..

[26] Luo Y., Zhang B., Cheng W.-H., Wang Q. (2010). Preparation, characterization and evaluation of selenite-loaded chitosan/TPP nanoparticles with or without zein coating. Carbohydr. Polym..

[27] Jang K.-I., Lee H.G. (2008). Stability of chitosan nanoparticles for l-ascorbic acid during heat treatment in aqueous solution. J. Agric. Food Chem..

[28] Yoksan R., Jirawutthiwongchai J., Arpo K. (2010). Encapsulation of ascorbyl palmitate in chitosan nanoparticles by oil-in-water emulsion and ionic gelation processes. Colloids Surf. B Biointerfaces.

[29] Bao S., Xu S., Wang Z. (2009). Antioxidant activity and properties of gelatin films incorporated with tea polyphenol-loaded chitosan nanoparticles. J. Sci. Food Agric..

[30] Dudhani A.R., Kosaraju S.L. (2010). Bioadhesive chitosan nanoparticles: Preparation and characterization. Carbohydr. Polym..

[31] Hu B., Pan C., Sun Y., Hou Z., Ye H., Hu B., Zeng X. (2008). Optimization of fabrication parameters to produce chitosan-tripolyphosphate nanoparticles for delivery of tea catechins. J. Agric. Food Chem..

[32] Zhang Y., Yang Y., Tang K., Hu X., Zou G. (2008). Physicochemical characterization and antioxidant activity of quercetin-loaded chitosan nanoparticles. J. Appl. Polym. Sci..

[33] Konecsni K., Low N.H., Nickerson M.T. (2012). Chitosan–tripolyphosphate submicron particles as the carrier of entrapped rutin. Food Chem..

[34] Keawchaoon L., Yoksan R. (2011). Preparation, characterization and in vitro release study of carvacrol-loaded chitosan nanoparticles. Colloids Surf. B Biointerfaces.

[35] Hadidi M., Pouramin S., Adinepour F., Haghani S., Jafari S.M. (2020). Chitosan nanoparticles loaded with clove essential oil: Characterization, antioxidant and antibacterial activities. Carbohydr. Polym..

[36] Mwangi W.W., Ho K.-W., Ooi C.-W., Tey B.-T., Chan E.-S. (2016). Facile method for forming ionically cross-linked chitosan microcapsules from Pickering emulsion templates. Food Hydrocoll..

[37] Tian H., Lu Z., Yu H., Chen C., Hu J. (2019). Fabrication and characterization of citral-loaded oil-in-water Pickering emulsions stabilized by chitosan-tripolyphosphate particles. Food Funct..

[38] Alehosseini E., Jafari S.M., Shahiri Tabarestani H. (2021). Production of d-limonene-loaded Pickering emulsions stabilized by chitosan nanoparticles. Food Chem..

[39] Sharkawy A., Barreiro M.F., Rodrigues A.E. (2020). Chitosan-based Pickering emulsions and their applications: A review. Carbohydr. Polym..

[40] Wu J., Ma G.-H. (2016). Recent Studies of Pickering Emulsions: Particles Make the Difference. Small.

[41] Wei Z., Wang C., Zou S., Liu H., Tong Z. (2012). Chitosan nanoparticles as particular emulsifier for preparation of novel pH-responsive Pickering emulsions and PLGA microcapsules. Polymer.

[42] Gan Q., Wang T., Cochrane C., McCarron P. (2005). Modulation of surface charge, particle size and morphological properties of chitosan–TPP nanoparticles intended for gene delivery. Colloids Surf. B Biointerfaces.

[43] Morris G.A., Castile J., Smith A., Adams G.G., Harding S.E. (2011). The effect of prolonged storage at different temperatures on the particle size distribution of tripolyphosphate (TPP)—Chitosan nanoparticles. Carbohydr. Polym..

[44] Jonassen H., Kjøniksen A.-L., Hiorth M. (2012). Effects of ionic strength on the size and compactness of chitosan nanoparticles. Colloid Polym. Sci..

[45] Bugnicourt L., Alcouffe P., Ladavière C. (2014). Elaboration of chitosan nanoparticles: Favorable impact of a mild thermal treatment to obtain finely divided, spherical, and colloidally stable objects. Colloids Surf. A Physicochem. Eng. Asp..

[46] Calvo P., Remunan-Lopez C., Vila-Jato J.L., Alonso M. (1997). Novel hydrophilic chitosan-polyethylene oxide nanoparticles as protein carriers. J. Appl. Polym. Sci..

[47] Sargazi G., Afzali D., Mostafavi A., Shadman A., Rezaee B., Zarrintaj P., Saeb M.R., Ramakrishna S., Mozafari M. (2019). Chitosan/polyvinyl alcohol nanofibrous membranes: Towards green super-adsorbents for toxic gases. Heliyon.

[48] Kaloti M., Bohidar H.B. (2010). Kinetics of coacervation transition versus nanoparticle formation in chitosan–sodium tripolyphosphate solutions. Colloids Surf. B Biointerfaces.

[49] Assadpour E., Jafari S.M. (2019). A systematic review on nanoencapsulation of food bioactive ingredients and nutraceuticals by various nanocarriers. Crit. Rev. Food Sci. Nutr..

[50] Rosyada A., Sunarharum W.B., Waziiroh E. (2019). Characterization of chitosan nanoparticles as an edible coating material. IOP Conf. Ser. Earth Environ. Sci..

[51] Bhattacharjee S. (2016). DLS and zeta potential—What they are and what they are not?. J. Control. Release.

[52] Ali M.E.A., Aboelfadl M.M.S., Selim A.M., Khalil H.F., Elkady G.M. (2018). Chitosan nanoparticles extracted from shrimp shells, application for removal of Fe (II) and Mn (II) from aqueous phases. Sep. Sci. Technol..

[53] de Carvalho F.G., Magalhães T.C., Teixeira N.M., Gondim B.L.C., Carlo H.L., dos Santos R.L., de Oliveira A.R., Denadai Â.M.L. (2019). Synthesis and characterization of TPP/chitosan nanoparticles: Colloidal mechanism of reaction and antifungal effect on C. albicans biofilm formation. Mater. Sci. Eng..

[54] Anand M., Sathyapriya P., Maruthupandy M., Hameedha Beevi A. (2018). Synthesis of chitosan nanoparticles by TPP and their potential mosquito larvicidal application. Front. Lab. Med..

[55] Sorrentino A., Gorrasi G., Vittoria V. (2007). Potential perspectives of bio-nanocomposites for food packaging applications. Trends Food Sci. Technol..

[56] Luo Y., Zhang B., Whent M., Yu L., Wang Q. (2011). Preparation and characterization of zein/chitosan complex for encapsulation of α-tocopherol, and its in vitro controlled release study. Colloids Surf. B Biointerfaces.

[57] Ali S.W., Rajendran S., Joshi M. (2011). Synthesis and characterization of chitosan and silver loaded chitosan nanoparticles for bioactive polyester. Carbohydr. Polym..

[58] Campos J., Díaz-García P., Montava I., Bonet-Aracil M., Bou-Belda E. (2017). Chitosan pretreatment for cotton dyeing with black tea. Proc. IOP Conf. Ser. Mater. Sci. Eng..

[59] Mihaela Predescu A., Matei E., Râpă M., Pantilimon C., Coman G., Savin S., Elisabeta Popa E., Predescu C. (2019). Adsorption of lead(II) from aqueous solution using chitosan and polyvinyl alcohol blends. Anal. Lett..

[60] Xiao Z., Wang E., Zhu G., Zhou R., Niu Y. (2016). Preparation, characterization and rheological behavior of chitosan nanocapsule emulsion encapsulated tuberose fragrance. Pol. J. Chem. Technol..

[61] Sivakami M.S., Gomathi T., Venkatesan J., Jeong H.-S., Kim S.-K., Sudha P.N. (2013). Preparation and characterization of nano chitosan for treatment wastewaters. Int. J. Biol. Macromol..

[62] Stulzer H.K., Tagliari M.P., Parize A.L., Silva M.A.S., Laranjeira M.C.M. (2009). Evaluation of cross-linked chitosan microparticles containing acyclovir obtained by spray-drying. Mater. Sci. Eng. C.

[63] Jingou J., Shilei H., Weiqi L., Danjun W., Tengfei W., Yi X. (2011). Preparation, characterization of hydrophilic and hydrophobic drug in combine loaded chitosan/cyclodextrin nanoparticles and in vitro release study. Colloids Surf. B Biointerfaces.

[64] Karimi M.H., Mahdavinia G.R., Massoumi B. (2018). pH-controlled sunitinib anticancer release from magnetic chitosan nanoparticles crosslinked with κ-carrageenan. Mater. Sci. Eng. C.

